# The Spectrum of Neurological Manifestations of Varicella–Zoster Virus Reactivation

**DOI:** 10.3390/v15081663

**Published:** 2023-07-30

**Authors:** Peter G. E. Kennedy

**Affiliations:** School of Psychology and Neuroscience, College of Medical, Veterinary & Life Sciences, Garscube Campus, University of Glasgow, Glasgow G61 1QH, Scotland, UK; peter.kennedy@glasgow.ac.uk

**Keywords:** herpes zoster, virus, latency, neuron, neurology, reactivation, Varicella–Zoster virus (VZV), vasculitis, post-herpetic neuralgia (PHN)

## Abstract

Varicella–Zoster virus (VZV) is a pathogenic human alpha herpes virus that causes varicella (chicken pox) as a primary infection and, following a variable period of latency in different ganglionic neurons, it reactivates to produce herpes zoster (shingles). The focus of this review is on the wide spectrum of the possible neurological manifestations of VZV reactivation. While the most frequent reactivation syndrome is herpes zoster, this may be followed by the serious and painful post-herpetic neuralgia (PHN) and by many other neurological conditions. Prominent among these conditions is a VZV vasculopathy, but the role of VZV in causing giant cell arteritis (GCA) is currently controversial. VZV reactivation can also cause segmental motor weakness, myelitis, cranial nerve syndromes, Guillain–Barre syndrome, meningoencephalitis, and zoster sine herpete, where a neurological syndrome occurs in the absence of the zoster rash. The field is complicated by the relatively few cases of neurological complications described and by the issue of causation when a neurological condition is not manifest at the same time as the zoster rash.

## 1. Introduction: The Problem of VZV Reactivation

Varicella–Zoster virus (VZV) is a double-stranded DNA alpha herpes virus that causes chicken pox (also known as varicella), generally in children as a primary infection, and subsequently causes herpes zoster (shingles) in adults. VZV is pathogenic in humans, and after the primary infection the virus becomes latent throughout the entire neuroaxis, where it is located primarily in neurons in human ganglia [1,2], including the dorsal root ganglia (DRG), trigeminal ganglia (TG), and autonomic ganglia, including those located in the enteric nervous system [3]. After a highly variable period, which may last for several decades, the virus may reactivate from the latent state to cause the extremely painful condition called herpes zoster, which is a vesicular skin eruption that occurs in one or more sensory dermatomes [3]. While herpes zoster is the most frequent and important manifestation of VZV reactivation from latency, it has been increasingly recognised that there is a wide spectrum of neurological complications that may follow VZV reactivation. In this overview, the focus will be on VZV reactivation rather than on the primary infection of varicella, the neurological aspects of which are covered elsewhere [1].

VZV has a genome of approximately 125,000 base pairs and there are 68 unique open reading frames (ORF) [4]. A great deal has been learned about VZV latency over the last few years, but this information will not be discussed in any detail here. While it is known that viral gene transcription during latency is highly restricted, it is now recognised that it is even more restricted than previously thought. Thus, though earlier studies demonstrated the transcription of VZV genes 21, 29, 62, 63, and 66 [5,6,7], a more recent study [8] using advanced deep-sequencing technology detected, convincingly, a unique spliced latency-associated VZV transcript in human TG neurons that maps anti-sense to the viral transactivator gene 61. That study analysed TG within 6 h of death, making viral reactivation following the death of the individual most unlikely, whereas many previous studies had analysed human ganglia around 24 h after death. This important observation was followed by another study [9] that reported the induction of a VLT-ORF63 fusion transcript that stimulated viral gene expression during VZV reactivation from latency. The previous detection by several groups of ORF63 in TG, obtained at autopsy, may well reflect detection of this VLT-ORF63 fusion [9,10]. Detailed discussions of viral gene expression during latency, the epigenetic control of VZV latency, and key immunological aspects of viral latency are provided elsewhere [10].

It should be appreciated that many of the published reports of VZV reactivation causing different neurological complications were based on just a few cases—sometimes just a series of case reports—and an unequivocal cause-and-effect relationship between VZV and the neurological syndrome cannot necessarily be established. Thus, there may be an association, or even a correlation, rather than a definite causation. However, when a VZV manifestation such as herpes zoster and the neurological phenotype are closely related in time and/or space, then a direct causation is far more likely and easier to justify. While there are reasonably large patient numbers in cases of VZV-induced vasculitis reports, this is not the case in general, and the small numbers of reported cases of many different neurological syndromes should always be borne in mind. The same paucity of numbers also has relevance to the treatment of many of these syndromes. However, where there is direct evidence of VZV involvement in a post-reactivation neurological complication, causation is easier to determine, although it should always be borne in mind that the apparent viral involvement may actually reflect an ongoing acute inflammatory process with non-specific viral reactivation.

## 2. The Role of Immunosuppression in VZV Reactivation

Different types of immunodeficiency states can predispose to VZV reactivation. These states can be due to primary or secondary immunodeficiencies (for a detailed review, see [11]). Several primary immundeficiencies can increase the likelihood of VZV reactivation, including herpes zoster and central nervous system (CNS) involvement, with possible defects in T cells, B cells, and natural killer (NK) cells [11]. Examples include severe combined immunodeficiency (SCID) and STAT5B deficiency [12] affecting T cells and NK cells with impaired interferon (IFN) α responses [11]. An important recent finding was that the innate cytosolic DNA sensor POL III (RNA polymerase III) predisposes to VZV infection in children and adults, including VZV vasculitis in identical twins [13]. In the latter study, the authors suspected a genetic aetiology of the recurrent VZV vasculitis, because of the unusual presentation in both monozygotic twins.

There are several types of secondary immunosuppression that may predispose to VZV reactivation syndromes. The most obvious type is the increasing incidence of VZV reactivation, particularly herpes zoster, with advancing age (see below), which is thought to be due to decreasing cell-mediated immunity to VZV as an individual gets older [1,3]. Patients with human immunodeficiency virus (HIV) infection have a specific CD4+ T cell defect, and this type of secondary immunodeficiency is associated with an increased risk of VZV reactivation, especially herpes zoster [11]. A broader immunosuppression due, for example, to malignant disease, chemotherapy, and organ transplantation, can also lead to VZV reactivation. Other secondary immunosuppressive drugs provided for a variety of autoimmune and other chronic conditions can cause VZV reactivation. Such drugs include, for example, Methotrexate, Natalizumab, and Tumour Necrosis Factor (TNF) alpha blockers. It should be appreciated that while intact cellular immunity to VZV appears to be critical in preventing VZV reactivation, the precise immunological mechanisms of this protection remain very poorly understood.

## 3. Herpes Zoster

Herpes zoster is the most frequent consequence of reactivation of latent VZV, but its precise incidence is difficult to quantify with precision, due in part to the fact that not all cases, especially mild ones, are reported. Early estimates suggested that it had an incidence of 3.4 per thousand in the UK [14] and 1.3 per thousand in an American population [15]. More recent analyses suggest an incidence in the UK of 2–3 per thousand patient years in individuals over 50 years, and 8 per thousand patient years in people aged 70 and over [16], clearly showing that zoster incidence increases with age. A recent study in a US population [17] reported that there was an age-specific transition of herpes zoster, with ongoing increases among younger adults, but deceleration in older adults. The reasons for this are unclear, but it may be related in part to VZV vaccination (see below). It was estimated that overall there were one million episodes of herpes zoster annually in the US [17]. By the age of 85 years, more than half of the population has reported at least one episode of zoster [3,18].

The most common site of herpes zoster is one of the thoracic nerves, followed by the ophthalmic division (V1) of the trigeminal nerve [16]. The latter is known as herpes zoster ophthalmicus and can lead to severe local eye complications. The seventh cranial nerve may also be a site of VZV reactivation, and when it is associated with a facial paralysis and otic zoster it is called the Ramsay–Hunt syndrome [19]. In the latter case, there are characteristic vesicles in the ear that may be accompanied by defects in hearing, as well as by vestibular symptoms [16]. The most important complication of herpes zoster is post-herpetic neuralgia (PHN), which is discussed below. The author can testify to the relentless and severe burning pain of herpes zoster and would advocate a course of Famciclovir at 750 mg per day for 7 days to commence within 72 h of the rash, as this has been shown to reduce the severity of the pain and the subsequent development of PHN. A course of oral Valaciclovir could also be given. It has also been suggested that, in patients with acute zoster, a combination of an oral corticosteroid (prednisolone) and an antiviral such as acyclovir should be considered, as this may reduce the acute pain and speed up the rate of healing of the painful skin lesions [16]. A detailed discussion of potential therapies for acute zoster is provided elsewhere [16].

## 4. Post-Herpetic Neuralgia (PHN)

The most frequent and important neurological complication of herpes zoster is PHN, which can be defined as pain that persists for three months or more after the onset of the zoster rash [1,3]. Approximately 50% of zoster patients over 60 years of age will develop PHN to some extent [19]. Predisposing factors for the development of PHN, apart from the obvious factors such as increasing age and immunosuppression causing patients to be more likely to develop zoster, include a severe or disseminated zoster rash, the presence of a prodrome, female gender, severe pain at the onset of the rash, and the presence of detectable VZV using the polymerase chain reaction (PCR) [16]. The pain of PHN can be extremely severe, even driving some individuals with relentless pain to suicide. Accordingly, PHN should be regarded with great seriousness. A variety of physical and drug treatments have been tried in cases of PHN (reviewed in [16]), including tricyclic antidepressants, anticonvulsants such as gabapentin and pregabalin, the application of local lidocaine patches, corticosteroids, and transcutaneous electrical nerve stimulation (TENS), but often the various treatments are ineffective at pain relief and, unfortunately, PHN can remain refractory to all treatments.

The cause of PHN remains unknown. In general terms, it has been suggested that there is an alteration of the electrical activity of ganglionic or spinal neurons following recovery from herpes zoster, or in these patients VZV exists in a persistent form in the affected ganglia [1,3,20]. Potentially, both of these potential mechanisms of PHN may be accurate. While most attention in PHN has focused on alteration by the virus of host factors, it is also possible that the strain of VZV itself may play a role in producing in PHN. Thus, it has been reported that in an in vitro system using cultured neuroblastoma cells, VZV obtained from PHN patients induced sodium current density increases, unlike VZV obtained from zoster patients who did not develop PHN [21]. Since alteration of voltage-gated sodium channels, leading to altered excitability, is associated with pain sensation, this is another potential mechanism of PHN.

It is possible, to some extent, to prevent both zoster and PHN. In a landmark trial [22] known as the shingles prevention study, a live attenuated VZV vaccine called Zostavax was provided in a placebo-controlled and double-blind manner to over 19,000 subjects and controls. It was found that immunised individuals showed a 51.3% reduction in the incidence of zoster, with a 63.9% reduction in the 60–69-year age group. Furthermore, the overall incidence of PHN was reduced in immunised individuals by 65% [1,3,22]. Importantly, the vaccine also reduced by over 31% the incidence of PHN in individuals who developed zoster, with the particularly vulnerable >70 age group showing the greatest benefit [22]. These results were certainly impressive, but the role of this vaccine in preventing zoster and PHN in immunocompromised and very old (>85 years) individuals was not clear. However, a more recently developed subunit VZV vaccine called Shingrix has been shown to promote both adaptive cellular immunity and innate immunity to VZV, and it is highly effective in reducing the incidence of zoster and PHN by 97% in both healthy individuals and those aged 70 or older [3,23]. Since this vaccine does not contain live virus, it can be administered to immunosuppressed individuals and much older individuals.

## 5. VZV Vasculopathy

It has been increasingly recognised that VZV reactivation can result in a vasculopathy in which there is a productive viral infection of cerebral vessels. Many of the insights regarding this condition have been provided by the neurology group at the University of Colorado School of Medicine, formerly headed by Donald Gilden. This complication is not surprising, since herpes zoster itself is associated with an increased risk of stroke [24]. VZV vasculopathy affects both small and large cerebral arteries in which there can be detected inflammatory infiltrations consisting of macrophages and T cells, as well as subsequent evidence of VZV DNA, VZV antigens, and characteristic histological evidence of a viral infection, such as multinucleate giant cells and inclusion bodies [1,24]. In addition, both the dural sinuses and extracranial vessels can be affected [24]. It has been pointed out that the spectrum of VZV vasculopathy is now very wide, as it can also produce cerebral aneurysms, arterial dissection, cerebral haemorrhage, transient ischaemic attacks, cerebral dural sinus thrombosis, spinal cord infarction, and peripheral thrombosis [24]. Thus, the syndrome is much greater than the classical presentation of a contralateral hemiplegia following an episode of herpes zoster ophthalmicus. The involvement of temporal arteries is discussed below.

The clinical presentation of VZV vasculopathy is highly variable, with typical symptoms such as focal features, increasing impairment of cognitive abilities, and seizures occurring at greatly varying periods after the initial rash, or even in the absence of a rash [18,24]. A diagnosis of cerebral vasculopathy may be suggested following neuro-radiological investigations such as computerised tomography (CT) and/or magnetic resonance imaging (MRI) scanning, which may show white-grey matter junction changes and ischaemic or haemorrhagic changes [3,24]. Arteriography may show characteristic vasculitic changes, including narrowing and/or beading of cerebral arteries [24,25] Although the diagnosis is usually made by demonstrating VZV in the cerebrospinal fluid (CSF) by PCR, a more sensitive method of detection of a VZV vasculopathy is the CSF presence of anti-VZV IgG (which is not present in healthy individuals), and only when the CSF is negative for both VZV DNA and anti-VZV IgG antibody can a diagnosis of VZV vasculopathy be definitely excluded [26]. Treatment is based on relatively few cases, but it seems reasonable to treat VZV vasculopathy in its various forms with a 1- to 2-week course of oral corticosteroids, together with a 2-week course of intravenous acyclovir.

## 6. Giant Cell Arteritis

Giant cell arteritis (GCA) (also known as temporal arteritis) should be treated as a medical emergency. Characterised pathologically as an inflammation in the vessel wall of the temporal arteries, it can cause sudden and irreversible blindness [25]. This latter complication makes it essential that prompt treatment with corticosteroids is started as soon as the diagnosis is confirmed or strongly suspected. Suspecting that GCA may be a form of VZV vasculitis, Gilden and colleagues looked for evidence of VZV antigens in the temporal arteries of patients with GCA, obtained post-mortem, both biopsy-positive and biopsy-negative [27,28]. Previous attempts to demonstrate VZV antigens in temporal arteries of GCA patients were negative [29,30]. In an important study, Gilden et al. [27] examined multiple tissue sections from a large number (82) of biopsy-positive GCA temporal arteries and reported that as many as 74% of these contained VZV antigens. It was also found that 40% of GCA-positive and VZV-antigen-positive temporal arteries contained VZV DNA, despite formalin fixation. Soon afterwards, this group showed that 64% of biopsy-negative but clinically proven cases of GCA (and also 22% of normal temporal arteries) contained VZV antigens [28]. These results suggest that patients with GCA who have VZV antigens in their temporal arteries should be treated with both corticosteroids and an antiviral such as acyclovir. However, these interesting results have not yet been replicated. Thus, a subsequent study (whose co-authors included one who had been on the original positive study) reported that only 12% (3 of 25 cases) of biopsy-proven GCA cases were positive for VZV antigens, and there was evidence of false positivity of VZV-antigen staining in several of the temporal artery biopsy tissues [31]. However, it is important to consider the relative numbers of samples analysed in these reports. Whereas Gilden et al. [27] studied 82 patient biopsies, Buckingham et al. [31] studied 25 patient biopsies. In addition, Gilden et al. cut 100 tissue sections from each biopsy and then analysed 50 alternate sections from these. It is this large number of tissue sections analysed in each case that may need to be analysed to obtain a positive VZV result in the GCA cases. It is also important to calculate an “n: value that is based on individual patients and not based on positive slides from a single biopsy. It is important that the different studies have similar scientific criteria for positivity.

The problem of false positive immunohistochemical detection of VZV antigens in these temporal arteries, due to antibody cross-reactivity, was highlighted in another study [32]. Further, it is not certain that direct causality has been demonstrated, as it is possible that VZV reactivation could result from the inflammatory process per se of GCA [33], although the authors thought that there was a cause-and-effect process, in that the VZV triggered the immunopathology [27].

In the author’s view, further studies and more data are required in order to prove that VZV causes GCA, and until that happens, it seems reasonable to treat GCA with corticosteroids alone, as is usually the case at present. Probably the only way to be absolutely certain of the efficacy of providing such patients with a combination of corticosteroids and acyclovir is to carry out a clinical trial of corticosteroids plus acyclovir versus corticosteroids alone in GCA, but it would be very difficult to justify such a trial until the presence of VZV in a large percentage of temporal arteries from biopsy-proven GCA patients has been established unequivocally. This ongoing controversy has been discussed in detail in a comprehensive review [34].

## 7. Encephalitis

In rare cases (probably complicating about 0.25% of all zoster cases), there may be meningoencephalitis, but this may be an underestimate, as the meningoencephalitis may be mild and even go unreported [1,19]. While it has been suggested that VZV encephalitis may be a form of vasculitis [35], it seems highly likely that VZV meningoencephalitis is a real and separate entity. The presentation is similar to most cases of viral encephalitis, with confusion, headache, fever, and meningeal symptoms. Although this complication is usually self-limiting and not serious, it may be more severe in an immunosuppressed patient. There is typically a CSF pleocytosis, and the CSF is also positive for VZV DNA by PCR and may contain the anti-VZV IgG antibody. While there is no evidence for particular treatments, it seems reasonable to treat such patents with oral corticosteroids for 7 days, with a 10-day to 14-day course of intravenous acyclovir.

## 8. Segmental Motor Weakness

It has been recognised for some time that zoster may be closely associated with segmental weakness. While this is an important complication, the pathogenesis is poorly understood. Typically, the motor weakness develops about 2 weeks following the zoster rash, but the interval is variable, ranging from 1 day to as long as 4 months [19], with cephalic zoster having a shorter interval. While there is usually a correlation between the dermatomal rash and the motor weakness, this is not always the case, and there may be a topographic dissociation between these in around 10% of case [19]. The latter phenomenon may be reinforced by the detection of electromyogram (EMG) changes in regions unaffected by the zoster rash. The motor weakness may also affect the intercostal muscles and diaphragm, but these may often be missed on examination unless specifically looked for. While the prognosis for VZV-associated motor weakness is generally thought to be quite good, with about a 50% recovery rate [3], most of the patients with this complication seen by the author have been left with some residual weakness. Motor weakness may also result from a VZV myelitis occurring 1–2 weeks after the zoster rash, typically causing bilateral leg weakness, sphincter disturbance, and a CSF pleocytosis, with characteristic MRI changes in the affected region of the spinal cord, such as cord swelling and hyperintensiites on T2 imaging [1,25]. While the diagnosis of segmental motor weakness is usually made clinically, the diagnosis can be established in myelitis by detecting VZV DNA in the CSF by PCR. In both cases, there is little evidence-based treatment, but it seems reasonable to treat these with a 10-day to 14-day course of intravenous acyclovir and at least a week’s course of oral corticosteroids.

## 9. Zoster Sine Herpete

It is now recognised that the neurological complications of VZV reactivation can manifest themselves in the absence of the typical herpes zoster rash. This presentation is known as zoster sine herpete (ZSH). This phenomenon was highlighted, in particular, by Gilden and colleagues, who described the cases of two individuals with dermatomal pain who had VZV DNA detected in their CSF [36]. The range of neurological conditions presenting in this way continues to increase, such that it is now accepted that zoster sine herpete should be considered in any patient with undiagnosed acute, subacute, or chronic cerebral or spinal-cord disease—in particular, if these patients also have a CSF pleocytosis [37]. The best method of confirming the diagnosis of ZSH in these individuals is to demonstrate VZV DNA in the CSF or peripheral blood mononuclear cells and/or CSF anti-VZV IgG antibody [24,37]. Such cases should probably be treated with a 10-day to14-day course of intravenous acyclovir and one week of oral corticosteroids.

## 10. Cranial Neuropathies

The propensity of reactivated VZV to affect the first division of the fifth (trigeminal) cranial nerve to produce herpes zoster ophthalmicus has been mentioned above, and this may sometimes be followed by a variety of serious local ophthalmic complications, such as scleritis, iritis, keratitis, and retinitis [1,3]. The involvement of the seventh (facial) cranial nerve to produce a facial palsy, which is termed the Ramsay–Hunt syndrome when accompanied by otic zoster, has also been noted above. Although these are, in some cases, based on case reports, most of the cranial nerves have been reported as being affected by herpes zoster [19], and in some cases they may be affected together.

## 11. Other Neurological Manifestations

There appears to be an association between the Guillain–Barre syndrome (GBS) and preceding herpes zoster. While there have been a number of case reports of this association, causation continues to be an issue, since both conditions are commonly seen by neurologists. The time interval between the initial zoster rash and the development of the inflammatory polyradiculitis varies considerably and may range from 2 days to several months [19]. The clinical presentation is the same as for usual cases of GBS, and a shorter interval (<2 weeks) between the rash and the GBS is associated with a worse prognosis [38]. Treatment of this presumably immune-mediated complication is the same as for GBS from other causes.

Several years ago, there was described a multifocal leucoencephalitis syndrome in two patients with malignant disease [39]. Zoster in the cervical and thoracic dermatomes in these individuals was followed by a progressive neurological condition that resembled the well-recognised condition of progressive multifocal leukoencephalopathy. These conditions can be seen as opportunistic infections, in which VZV is able to directly infect brain cells such as glial cells and neurons [19].

## Data Availability

Not applicable.
